# Nest founding by mixed kin groups in communally nesting orchid bees

**DOI:** 10.1098/rsbl.2025.0003

**Published:** 2025-07-16

**Authors:** Jonas Henske, Mauricio Fernández Otárola, Janosch Dohrs, Thomas Eltz

**Affiliations:** ^1^ Animal Ecology, Evolution and Biodiversity, Ruhr-Universitat Bochum, Bochum, North Rhine-Westphalia, Germany; ^2^ Center for Population Biology, University of California, Davis, CA, USA; ^3^ Biodiversity and Tropical Ecology Research Center, School of Biology, University of Costa Rica, San José, San José Province, Costa Rica

**Keywords:** Euglossini, social behaviour, microsatellites, *Euglossa cybelia*, communal nesting, nest founding, pleometrosis

## Abstract

Neotropical orchid bees are the only tribe within the corbiculate bees that does not exhibit obligate eusociality, making them an intriguing study group with regard to the evolution of social behaviour. However, finding nests is challenging, and nesting behaviour has been described only for a small fraction of the known species. Here, we present nests and kinship analyses for the aerially nesting species *Euglossa cybelia* in Pacific lowland Costa Rica*,* revealing a unique case of communal nest founding by multiple sister groups with no apparent social hierarchies. Within the studied nests all females were mated and the majority of foundresses contributed to the first generation offspring. We hypothesize that the elaborate nest architecture and effort associated with its construction have promoted co-founding and communal nesting in *E. cybelia*.

## 1. Background

Understanding how and why social behaviour evolves is a central question in evolutionary biology [1]. Social Hymenoptera offer an exceptional system to study the origins and transitions of sociality due to their wide spectrum of nesting strategies and social organizations [2]. These societies typically consist of closely related females, with reproduction concentrated in a dominant individual [3,4]. Although groups of unrelated individuals are regularly associated with cooperative breeding in vertebrates [5], this is rarely the case in Hymenoptera [6,7]. Studying close relatives of highly social Hymenoptera can provide insights into the factors driving the early evolution of social behaviour [8].

The neotropical orchid bees belong to the corbiculate bees (Apinae) and are currently believed to be the earliest-branching group in that clade [9–11]. In contrast to orchid bees, the three derived groups of the clade, honeybees, bumblebees and stingless bees, all exhibit obligate eusociality (or social parasitism in some bumblebees), suggesting a single origin of eusociality with no reversal to solitary living [9]. Therefore, orchid bees are a particularly interesting lineage in which to study the evolution of social behaviour [12]. Orchid bees are renowned for the pollination services they provide to a large number of tropical plants [13–17], thanks to the peculiar behaviour in which male bees collect exogenous volatiles from various sources [18]. These volatiles are used to concoct complex perfume blends [19,20], to attract females [21,22], possibly reflecting fitness-related traits [23]. However, in contrast to the well-studied males, little is known about the behaviour of females. Nests are difficult to find in the natural habitat, and therefore, nesting behaviour has been described only for approximately 20% of the known species [24,25].

Orchid bees have long been considered solitary, but an increasing number of studies show that social nesting exists in many species, at least facultatively (reviewed in [25]). Multiple cavity-nesting *Euglossa* spp*.* show a dominant–subordinate social hierarchy, with mothers (foundresses) being reproductively dominant over younger nest mates [26,27]. This is consistent with observations of oophagy in various species [28–34] resulting in reproductive skew in favour of dominant individuals [35,36]. Constant 24 h nest observations and transcriptomic analyses showed that *Euglossa dilemma* expresses four different categories/stages of solitary and social behaviours [37], with subordinate individuals being capable of expressing the same behavioural, physiological, genetic and chemical traits as seen in foundresses [38].

In other cavity-nesting *Euglossa* species [39–41] as well as in the genera *Eufriesea* (reviewed in [42]) and *Eulaema* [43], females were observed sharing a nest (cavity) but working independently on their own brood cell clusters suggesting communal nesting behaviour. From here onwards we refer to communal nesting behaviour as defined by Michener [44]: multiple females, related or unrelated, share nesting space while independently constructing, provisioning and ovipositing their own cells.

**Figure 1.**
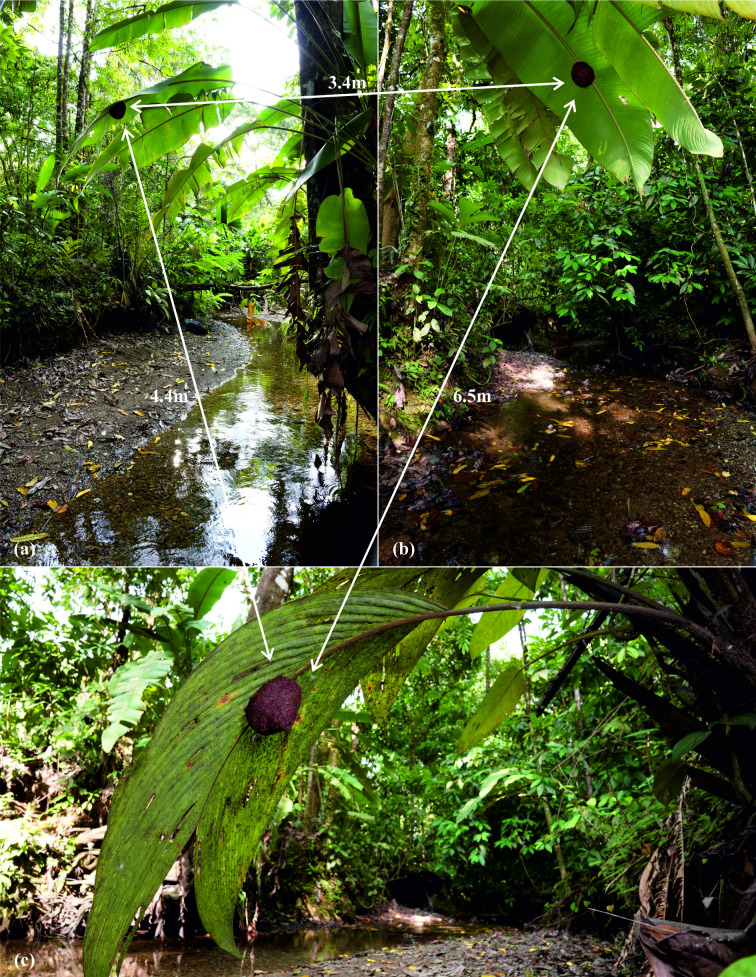
Nests found in 2020 at the riverbank of the Quebrada Negra at Tropical Field Station La Gamba. N1 (a) and N2 (b) were found under leaves of *Heliconia imbricata*, N3 (c) of *Asterogyne martiana*.

Aerially nesting orchid bee species are the least studied among orchid bees. Aerial nests are usually made from resin and attached to the undersides of leaves (figure 1) or stems. Multiple females have been observed in nests of some aerially nesting *Euglossa* species [45–48]. Notably, in these species, brood cells were always in close contact with each other, with no separate, independent clusters observed. Communal behaviour was postulated in some of these cases [45,46,48]. In both cavity and aerially nesting species, the assumption of communal behaviour was often based on incomplete behavioural observations or deduced from the absence of differences in mating status and ovary sizes [39–41,45,46,48]. The latter, however, is not sufficient to demonstrate the lack of reproductive skew: studies revealed that in species showing strong reproductive hierarchies, both dominant and subordinate individuals can be inseminated and can have similarly developed ovaries [27,33,37].

Notably, in almost all cases where euglossine multi-female nests were observed, nests were founded or re-activated by a single individual with additional females joining later. In one case, an existing old nest was re-activated by two females at about the same time [40]. In contrast, the founding of new nests by multiple females—rather than the reactivation of existing nests—has been only observed in one species, *E. cybelia*, where up to five individuals cooperated in the construction of a large resinous nest envelope prior to the construction of any brood cells [48]. Communal behaviour was presumed. However, the degree of relatedness of the foundresses and whether they were derived from a single source nest with pre-established hierarchies remained unknown. In this study, we provide new insights into the unique nest founding behaviour of *E. cybelia* by providing kinship analysis of individuals in six nests found in Pacific lowland Costa Rica. In addition, we included and analysed samples taken from a nest that was observed during the original study on nesting behaviour in *E. cybelia* by Solano-Brenes *et al.* [48].

## 2. Methods

The study took place in 2020 and 2023 at the Tropical Field Station La Gamba, Puntarenas, Golfo Dulce region, Costa Rica, which is located adjoining the Piedras Blancas National Park in the Pacific south of the country. The study area receives considerable annual precipitation (approximately equal to 6000 mm) and maintains consistently warm temperatures, averaging around 28°C [49]. We looked for nests of *E. cybelia* along small rivers on the property of the field station. We found two nests in close proximity to each other on 14 March 2020. Nest 1 (N1) showed a complete envelope (figure 1), whereas Nest 2 (N2) was still under construction. On 20 March 2020, we discovered an additional third nest (N3) close to the previously observed nests (figure 1). N1 and N2 were found under leaves of *Heliconia imbricata,* N3 under a leaf of *Asterogyne martiana*. These nests were 3.4, 4.4 and 6.5 m apart, respectively. On 20 April 2023, we found three nests (N4–N6) located in a different ravine approximately 400 m from the original cluster. These three nests were all attached to leaves of *Asterogyne martiana* palms located within a radius of 27 m with a minimum distance of 19 m between nests. All nests were located between 1 m and 3 m above the ground.

### (a) Nest activity

Nest activity was only observed in 2020. To assess interactions between nests, we marked four individuals in N1 on 15 March before construction of the envelope of N2 was finished. We also marked the only individual from N3 we had encountered on 22 March. For marking, we enclosed the nest with a nylon mesh at night. In the morning, we caught the departing females and marked them individually with numbered plastic tags (Opalith tags; Holtermann Imkereibedarf, Brockel, Germany). We did not mark females of N2 (see also electronic supplementary material, nest activity observations).

### (b) Sampling

All nests were sampled at night on 24 March 2020 and on 2 April 2023 and were each transferred to a separate insectary (40 × 40 × 60 cm; Aerarium). On the next day, we dissected spermathecae from the sampled adult females to examine mating status by visually checking for sperm cells under a microscope before transferring them to 95% alcohol for later DNA extraction. We counted closed brood cells and those that were still being provisioned. In 2020, we counted the number of parasitized brood cells and transferred all brood and parasites to 95% alcohol. In 2023, we let the bees emerge within the insectaries and transferred all emerged bees and parasites to 95% alcohol.

### (c) Kinship analysis

For kinship analysis, we analysed all sampled adult females (*n* = 32) and all offspring (*n* = 55; hatched imagoes, pupae, larvae and eggs) from the six nests in the study. Additionally, we analysed a subset of 18 individuals sampled during the previous study on *E. cybelia* [48]. These individuals were the first breeding cohort plus all adult males that had emerged from a newly constructed nest (in the following referred to as N7). We used 22 microsatellite markers (see electronic supplementary material, table S1, designing of markers, DNA extractions, PCR conditions).

We calculated Queller & Goodnight’s [50] pairwise relatedness coefficients (r) between adults in 2020 and 2023 and between adults and offspring per nest using the ‘related’ package in R (v. 4.4.1; see also electronic supplementary material, relatedness coefficient). Based on r, we identified the most likely full-sib clusters within and across nests per study year, assigned the offspring to the sampled mother individuals and identified the most likely full-sib clusters within the offspring in each nest. The assignments were verified by using the program COLONY ([51]; default settings; haplodiploidy; male polygamy; female monogamy) and by visually comparing alleles among individuals taking advantage of the haplodiploid reproduction system and the fact that orchid bee females mate only once in their lifetime [22,52].

## 3. Results

### (a) Nest activity

**Figure 2.**
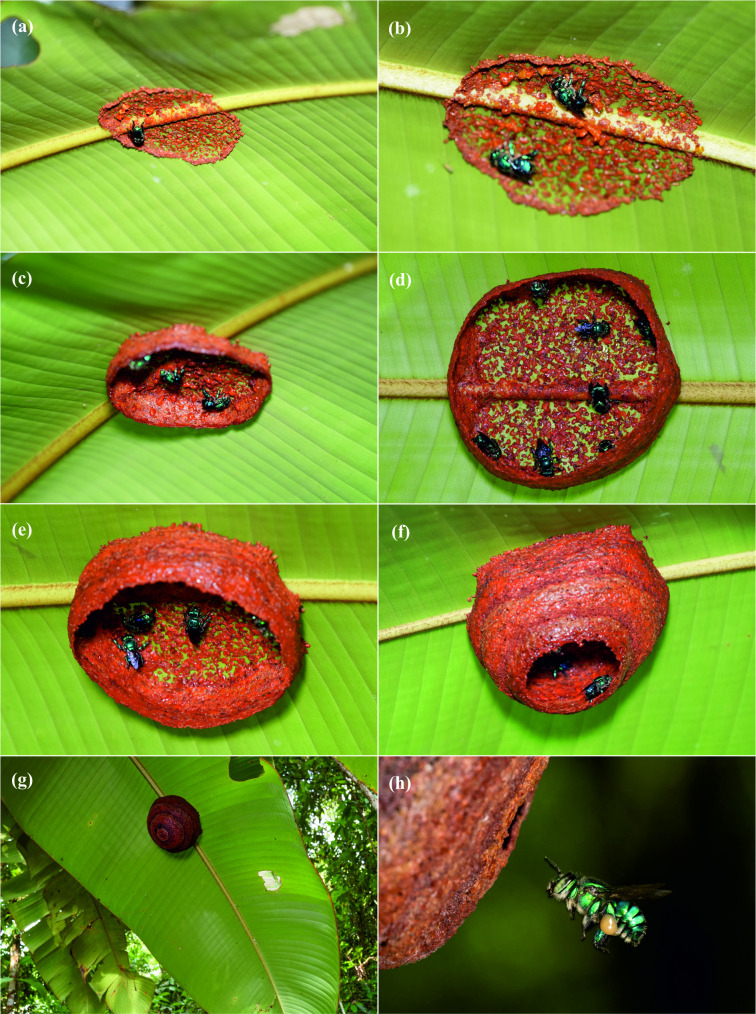
Early nest construction of group-nesting *Euglossa cybelia* at La Gamba, Costa Rica (nest N2). (a–f) Construction of the resinous base and nest envelope by multiple females prior to the construction of brood cells. A maximum number of seven females (d) were seen at the same time constructing the envelope. After 5 days, the envelope was completed (g). (h) Female *E. cybelia* with pollen load entering nest.

We observed up to seven females present at the same time during the constructing of the base and envelope of N2 (figure 2). Envelope construction took 5 days. We had marked four adult females in N1 on 17 March before the construction of the envelope of N2 was completed. On subsequent days, three of these females were seen at the nest entrance of N1 or returning to N1 from foraging trips. Notably, these marked females were never observed approaching or entering N2 and they did not contribute to the construction of its envelope. On 20 March, we found N3, and on 21 March, we observed one unmarked female exiting from N3 and entering N2 without any visible pollen or resin load. This was at a time when the envelope of N2 was already completed. After this sighting, we chose to mark all resident females in N3 but encountered only one individual. We marked this female and observed it leaving N2 on 23 March. When sampling all nests on 25 March, we did not find this individual (see also electronic supplementary material, nest activity).

### (b) Sampling

On average, we found six adult females (range: 2−16) and 23 cells (range: 14−30) per sampled nest, showing high variability in nest composition at the time of sampling (table 1).

**Table 1.** Sampling results of analysed nests. The number of encountered females in the parental generation (adults), as well as the number of cells with eggs, larvae, pupae or imagoes from the offspring generation, and parasites, are specified for each nest.

nest	adults	open brood cells		closed brood cells (2020) hatched individuals (2023)				
		eggs	empty	eggs	larvae	pupae	imagoes	parasites
N1 (2020)	3	3	8	0	7	0	0	7^a^
N2 (2020)	16	5	11	7	0	0	0	0
N3 (2020)	6	0	2	0	0	24^b^	4♀^c^	0
N4 (2023)	2	0	0	n/a	n/a	n/a	3♂, 4♀	7^a,d,e^
N5 (2023)	2	0	0	n/a	n/a	n/a	1♂^f^	0
N6 (2023)	4	0	1	n/a	n/a	n/a	5♂, 17♀	4^d^
N7 (2015)	n/a	n/a	n/a	n/a	n/a	n/a	18♂	0

^a^ Chalcidoidea; ^b^ all dead; ^c^ one dead; ^d^ Ichneumonidae; ^e^ Lepidoptera; ^f^ 19 closed brood cells were empty at final sampling.

Spermatheca dissections revealed sperm in all but three sampled females. All three females without sperm were found in N3. Two of them were probably recently emerged judging by the presence of two freshly eclosed brood cells in N3. The third female was parasitized by a large dipteran endoparasitoid larva, tentatively identified as Conopidae (see electronic supplementary material, figure S1) and we could not find the spermatheca.

### (c) Kinship analysis

**Figure 3.**
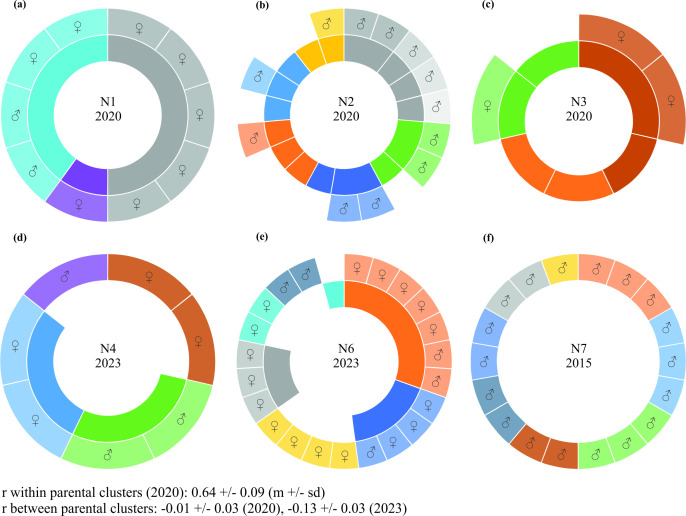
Kinship analysis for sampled adult females (parental generation, inner ring) and brood (offspring generation, outer ring) for N1 (a), N2 (b), N3 (c), N4 (d), N6 (e) and N7 (f). N5 is not shown because it contained only one female and one offspring. Each segment represents a single individual. See electronic supplementary material for pairwise relatedness histograms (figure S2) and raw data table for individual assignments (see data repository). (a–e) Saturated colours (inner ring) indicate most likely full-sib clusters based on Queller & Goodnight’s r within and across nests in 2020 and 2023, within parental generation. Graded colours (outer ring) indicate most likely offspring full-sib clusters and assignment to mother individuals based on r and visual allele inspection. Sex determination based on allele analysis (a–c) or based on visual checking of emerged bees (d–f) is given for the offspring generation (eggs, larvae or imagoes). (f) For N7 only the emerged offspring (all males) are shown; the parental generation was not sampled.

In 2020, kinship analysis of adult females revealed nine full-sib clusters across N1–N3 with two clusters consisting of only one individual (see figure 3 inner rings) and six different clusters in the newly founded nest N2 (r within clusters across nests = 0.64 ± 0.09 (GM ± SD), r between clusters = −0.02 ± 0.05). All adult females sampled in 2023 (N4–N6) were unrelated, i.e. no full-sibs (r = −0.12 ± 0.02; see also electronic supplementary material, figure S2 and raw data table in data repository).

Within a given nest the sampled offspring was assigned to three (N1), nine (N2), two (N3), four (N4), six (N6) and eight (N7) individual mothers or, in case of absent mothers, to full-sib clusters (see figure 3 outer rings). All the offspring in N2 were monomorphic (hemizygous) at all analysed markers, i.e. haploid males. All the offspring that emerged from N7 were also males. The offspring in N3 consisted only of females and in N1, N4 and N6 of both males and females.

## 4. Discussion

Reproductive division of labour is a hallmark of eusociality [53], yet it remains little studied in primitively social Hymenoptera. Euglossini—the closest relatives of highly eusocial honeybees, bumblebees and stingless bees—have been the subject of comparably few studies. Here, we present compelling evidence for elaborate communal nesting in *E. cybelia*, where multiple sibling groups co-found nests apparently without establishing a reproductive hierarchy, regardless of whether nests are newly constructed or longer established. The absence of reproductive skew stands in contrast not only to other orchid bee species, where dominant females typically monopolize reproduction [28–32,36,37], but also to other primitively social Hymenoptera, including small carpenter bees [54], plasterer bees [55], hover wasps [56] and social apoid wasps [57].

Nest co-founding by multiple females (pleometrosis) occurs in some primitively eusocial tropical *Polistes* paper wasps [58–60], but it is facultative [58], and brood production is typically monopolized by a single female over time [61,62]. In ground-nesting, communal *Perdita* bees (Andrenidae), nest co-founding may also occur [63,64], but solitary nesting with natal nest reuse seems to be more common [64,65]. Similarly, in the communal bee *Microthurge corumbae* (Megachilidae), multiple females reuse nests, but co-founding of new nests has not been documented [66]. The closest known analogue to the nesting biology of *E. cybelia* may be allodapine bees (*Exoneura s.s.*), where up to eight females may co-found a nest, exhibiting low reproductive skew [67–69]. However, co-founding is not obligatory and nest reuse is common [68,70,71]. Accordingly, in older reused nests reproduction becomes highly skewed, indicating semisocial nesting [72]. Thus, *E. cybelia* represents an unusual, if not unique, case of obligate co-founding in a communal bee. While Michener [44] did not explicitly address nest founding by multiple females in his definition of communal behaviour, our observations align with his core criteria: absence of reproductive skew and lack of cooperative brood care. Further studies are required to confirm the generality of our results.

The distinct nest architecture of *E. cybelia* might be an explanation for the unusual behaviour. The bees construct a large resinous envelope before building brood cells (see figure 2 and [48]). This process requires substantial time and energy, making cooperation among multiple females—whether related or not—particularly beneficial. In contrast, species that use pre-existing cavities may have less incentive to collaborate, leading to solitary founding or dominant-subordinate hierarchies [28–32,36,37]. However, not all observations support this pattern. In other aerially nesting species where multiple-female nests have been observed such as *E. hyacinthina* and the closely related *E. championi* [73]*,* resinous nest envelopes are constructed by a single female, with additional females joining later, if at all [45–47]. Notably, *E. hyacinthina* nests [74], though somewhat smaller than those of *E. cybelia* [48], still represent substantial constructions. Documentation of envelope construction in *E. hyacinthina* is limited, but in one case, a single female completed the remaining two-thirds of an envelope within 4 days (the initial stages were not recorded; [74]). The overall estimated construction time was 6 days, indicating that a single female can achieve envelope completion. However, the early stages of resin deposition may take significantly longer. From our observations and those of Solano-Brenes *et al.* [48] it is clear that *E. cybelia* nests are founded by surprisingly large numbers of females. In the present study, we found 16 adult females in a newly constructed nest, making it the largest nest of an aerially nesting *Euglossa* described to date. The large colony size may itself contribute to the low reproductive skew observed, as it becomes more difficult for dominant individuals to control subordinate females in larger groups—a pattern recently demonstrated in *E. dilemma* [75].

Notably, newly constructed nests (N2, N7) produced exclusively male offspring (see figure 3b,f), which, in Hymenoptera, develop from unfertilized eggs. In at least one studied nest (N2), this was not due to a lack of sperm, as all females were mated and therefore capable of producing female offspring. While our sample size is too small to draw firm conclusions, this observation may suggest that initial male-biased brood production could serve as a strategy to reduce competition, as daughters would otherwise compete with their mothers for resources and nesting space. In contrast, in species with social hierarchies, such competition does not occur. In *E. viridissima* [76] and *E. dilemma* (pers. obs.)*,* which exhibit social hierarchies, newly founded nests produce more female than male offspring.

The mechanisms by which foundresses gather to initiate envelope construction remain elusive. Some co-foundresses in our study likely originated from the local nest cluster (N3 or N1, figure 1). They may have come from N3, an adjacent nest discovered after envelope completion, or may have been unmarked females from N1. However, it seems unlikely that all co-foundresses originated from the local nest group, given the relatively low number of brood cells in those nests and the overall scarcity of observed interactions between nests. More distant nests within the same general area (watershed) were likely involved. The low number of observed interactions between nearby nests is somewhat surprising, as proximity and shared sociogenetic background should facilitate movement between natal and new nests, as seen in *Halictus* [77,78]. However, in *E. cybelia*, there is little evidence of individual females using multiple nests. Additionally, there seems to be no transition phase where an individual maintains contact between mother and daughter nests. In contrast to the closely related, strictly eusocial stingless bees [79], there is no transfer of nesting materials or food provisions between mother and daughter nests (see also [48]). The most plausible driver of founding a new nest may be that parasite pressure increases continuously over nest lifetime, supported by the observation that most of the older nests in our study had high levels of brood parasites (table 1). Additionally, older nests appear to lose structural stability as the resin becomes porous and brittle over time. Finally, the lifespan of the supporting leaf sets an upper limit on nest longevity. These factors likely prevent long-term nest reuse or reactivation, which is commonly observed in other *Euglossa* species [28,30,33]. In contrast to species where nests are reused across generations, the rather short nesting cycle of *E. cybelia* may instead favour the repeated founding of new nests. This, in turn, could promote co-founding by unrelated females and contribute to the observed low reproductive skew.

## Data Availability

Raw data and metadata are deposited at figshare and will be publicly available as of the date of publication [80]. This study does not report original code. Supplementary material is available online [81].
